# Detection of a biolistic delivery of fluorescent markers and CRISPR/Cas9 to the pollen tube

**DOI:** 10.1007/s00497-021-00418-z

**Published:** 2021-06-19

**Authors:** Shiori Nagahara, Tetsuya Higashiyama, Yoko Mizuta

**Affiliations:** 1 Institute of Transformative Bio-Molecules (WPI-ITbM), Nagoya University, Furo-cho, Chikusa-ku, Nagoya, Aichi 464-8601 Japan; 2 Division of Biological Sciences, Graduate School of Science, Nagoya University, Furo-cho, Chikusa-ku, Nagoya, Aichi 464-8602 Japan; 3 Department of Biological Sciences, Graduate School of Science, The University of Tokyo, 7-3-1 Hongo, Bukyo-ku, Tokyo, 113-0033 Japan; 4 Institute for Advanced Research (IAR), Nagoya University, Furo-cho, Chikusa-ku, Nagoya, Aichi 464-8601 Japan

**Keywords:** Pollen tube, Particle bombardment, CRISPR/Cas9, Fluorescent protein, *Nicotiana benthamiana*, *Nicotiana tabacum*

## Abstract

**Key message:**

***Biolistic delivery into pollen.***

**Abstract:**

In recent years, genome editing techniques, such as the CRISPR/Cas9 system, have been highlighted as a new approach to plant breeding. *Agrobacterium*-mediated transformation has been widely utilized to generate transgenic plants by introducing plasmid DNA containing CRISPR/Cas9 into plant cells. However, this method has general limitations, such as the limited host range of *Agrobacterium* and difficulties in tissue culture, including callus induction and regeneration. To avoid these issues, we developed a method to genetically modify germ cells without the need for *Agrobacterium*-mediated transfection and tissue culture using tobacco as a model. In this study, plasmid DNA containing sequences of Cas9, guide RNA, and fluorescent reporter was introduced into pollen using a biolistic delivery system. Based on the transient expression of fluorescent reporters, the *Arabidopsis UBQ10* promoter was found to be the most suitable promoter for driving the expression of the delivered gene in pollen tubes. We also evaluated the delivery efficiency in male germ cells in the pollen by expression of the introduced fluorescent marker. Mutations were detected in the target gene in the genomic DNA extracted from CRISPR/Cas9-introduced pollen tubes, but were not detected in the negative control. Bombarded pollen germinated pollen tubes and delivered their contents into the ovules in vivo. Although it is necessary to improve biolistic delivery efficiency and establish a method for the screening of genome-modified seeds, our findings provide important insights for the detection and production of genome-modified seeds by pollen biolistic delivery.

**Supplementary Information:**

The online version contains supplementary material available at 10.1007/s00497-021-00418-z.

## Introduction

Recently, rapid progress has been made in the development of genome engineering techniques, which have made it possible to perform specific modifications of selected target genes. One of the most widely used methods is targeted genome editing using clustered regularly interspaced short palindromic repeats (CRISPR) and CRISPR-associated protein 9 nuclease (Cas9) (Cong et al. 2013). This method is widely used in various organisms, including plants, because of its suitability for genetic engineering (Li et al. 2013; Nekrasov et al. 2013; Osakabe and Osakabe 2015; Osakabe et al. 2016). In animals, to produce genetically heritable traits of interest, Cas9 protein and sgRNA complexes are delivered into zygotes or eggs, resulting in the highly efficient production of genetically modified animals (Wang et al. 2013). In flowering plants, flowers contain male and female gametophytes that produce gametes. The female gametophyte of angiosperms is located in the ovary, and the female gamete (egg cell) is deeply embedded within the ovule (Zhou and Dresselhaus 2019). After flowering, pollen grains land on the stigma of the pistil, and they produce cylindrical tip-growing cells, referred to as pollen tubes, which contain two male gametes (sperm cells). Unlike animal cells, angiosperm sperm cells are non-motile; thus, pollen tubes play an essential role in conveying these cells toward the ovule enclosing an egg cell and a central cell to facilitate double fertilization (Dresselhaus et al. 2016). Pollen tubes serve as vector cells that deliver copies of the male genome to the female gametophyte to produce seeds that give rise to the next generation. Pollen is easier to access and handle than egg cells or zygotes, and such features are common to a wide range of angiosperms.

To generate genome-modified plants, it is necessary to introduce a CRISPR/Cas9 cassette into plant cells. Methods currently used to deliver materials to plant cells can be divided into three broad categories: chemical, physical, and biological (Newell 2000). Among the chemical methods, polyethylene glycol (PEG) can be used to facilitate highly efficient transfection (Toda et al. 2019); however, this approach requires the production of protoplasts, from which it is typically difficult to regenerate plants via callus, and tends to be associated with a high frequency of somaclonal variation (Fossi et al. 2019). Physical delivery methods, such as electroporation, also require the production of protoplasts to enable gene transfer (Woo et al. 2015). Recently, magnetic nanoparticles have been reported as novel physiological transformations (Zhao et al. 2017). This interesting method does not require protoplasts or tissue culture, but can only be applied in cotton so far (Vejlupkova et al. 2020). *Agrobacterium*-mediated transformation of foreign genes is the most frequently used biological technique in plants (Clough and Bent 1998); however, it has only been applied in a limited range of model plants, for which sufficient infection and culture methods have been established. Similarly, callus induction and/or plant regeneration is difficulties that arise during tissue culture in most plant species (Bregitzer et al. 1998). Therefore, the development of a simple and convenient method for introducing CRISPR/Cas9 into a wide range of plant species will contribute to significant advances in plant molecular genetic studies.

Biolistic delivery, also referred to as particle bombardment, can be used to facilitate the transient introduction of exogenous substances into cells (Sanford 2000). This technique has been widely used to transfer genes into cells and even into organelles (Okuzaki et al. 2013) in a range of plant species (Wang and Jiang 2011). Notably, in recent years, biolistic delivery has been applied to deliver the CRISPR/Cas9 cassette into immature maize embryos (Svitashev et al. 2016, 2015) and wheat embryos (Hamada et al. 2017). Given that embryos become mature plants, genetic manipulation of their cells can be effective. However, the generated embryos become chimeric plants, which necessitate subsequent selection in the resulting progenies.

To this end, in the present study, we developed a method for the biolistic delivery of CRISPR/Cas9-harboring plasmid DNAs into plant pollen to engineer the genome of male gametophytes and detection of cells having foreign genes using fluorescent markers. Pollen grains are simple structures containing male germ cells, which are suitable for the introduction of exogenous materials, as has been demonstrated recently with various approaches (Bhowmik et al. 2018; Eapen 2011; Zhao et al. 2017). We identified a suitable promoter for the control of gene expression in the pollen of four angiosperms following particle bombardment and assessed the efficiency with which genes were introduced into pollen using this technique. As a consequence of introducing plasmid DNA containing CRISPR/Cas9, we detected certain mutations within the target sequence in genomic DNA extracted from both leaves and pollen tubes. Additionally, we observed that bombarded pollen germinated and delivered generative or sperm cells that reached the ovules in vivo. These results will contribute to the production of genome-modified plants using a biolistic delivery system without *Agrobacterium* and tissue culture.

## Materials and methods

### Plant materials

The seeds of *Nicotiana tabacum* cv. ‘Petit Havana SR1’ were obtained from the Leaf Tobacco Research Center (Japan Tobacco Inc., Tochigi, Japan). *Nicotiana benthamiana*, *N. tabacum*, and *Torenia fournieri* cv. ‘Blue and White’ plants were grown in soil in a green room at 25–30 °C under long-day conditions (16-h light/8 h-dark), and *Solanum lycopersicum* cv. ‘Micro-Tom’ plants were grown in soil in a greenhouse at 20–30 °C. Young leaves and mature pollen from newly opened flowers were used for bombardment.

### Plasmid construction

The plasmid vectors used for particle bombardment in this study are listed in Table S1. The constructs *LAT52p::mApple* (YMv32) and *AtRPS5Ap::H2B-tdTomato* (DKv277) were obtained from previous studies (Adachi et al. 2011; Mizuta et al. 2015). *35Sp::H2B-mClover* (DKv700) and *35Sp::H2B-tdTomato* (DKv744) were provided by Dr. Daisuke Kurihara, and *35Sp::mTFP1* (DKv327) was provided by Dr. Noriko Inada. The *AtRPS5Ap::sGFP* vector (sSNv10), in which the *sGFP* gene was driven by the *Arabidopsis thaliana RPS5A* (*RIBOSOMAL PROTEIN SUBUNIT 5A; At3g11940*) promoter, was produced by inverse PCR of the *AtRPS5Ap::H2B-sGFP* vector (Maruyama et al. 2013) and self-ligation of the PCR product to remove the *H2B* (*HISTONE 2 B; At1g07790*) sequence. The sequences of *AtUBQ10p::sGFP* (sSNv25), *AtUBQ10p::tdTomato* (sSNv26), and *AtUBQ10p::H2B-mClover* (sSNv28) followed by the *Nos*-terminator sequence were isolated from the DKv909, DKv922, and DKv916 vectors, respectively (Kurihara et al. 2015), by *Hin*dIII/*Eco*RI digestion. Each fragment was cloned into the pGreen0029 vector (Hellens et al. 2000) using *Hin*dIII and *Eco*RI sites. The CRISPR/Cas9 vector targeting the *NbPDS3* gene (sSNv21) was constructed based on a previous study (Tsutsui and Higashiyama 2017). The *AtRPS5A* promoter was replaced with the *AtUBQ10* (*UBIQUITIN 10; At4g05320*) promoter to drive Cas9 gene expression, and the target sequence for the *NbPDS3* gene was then introduced via *Aar*I digestion (sSNv21). Primers used for plasmid construction are listed in Table S2.

### Plant transformation

The sSNv28 vectors were introduced into *Agrobacterium tumefaciens* strain LBA4404 harboring the pSoup plasmid (Hellens et al. 2000) by electroporation, and *N. benthamiana* leaf disks were infected with *Agrobacterium*. The infected leaf disks were cultured on callus induction medium [1× Murashige and Skoog basal medium, 3% (w/v) sucrose, 0.8% (w/v) Bacto agar, adjusted to pH 5.8, with KOH] containing 0.05 mg/L 1-naphthaleneacetic acid, 0.5 mg/L 6-benzylaminopurine, 100 mg/L kanamycin sulfate, and 300 mg/L cefotaxime sodium. The plants that regenerated from calli were transferred to a medium lacking hormones to induce root germination and were eventually transferred to the soil.

### DAPI staining of pollen

Wild-type pollen grains were fixed with a 9:1 mixture of ethanol and acetic acid (v/v) for 10 min, and the samples were directly stained with DAPI solution and incubated for more than 10 min. After staining, the pollen grains were washed twice with water, mounted on glass slides, and observed under an inverted fluorescence microscope (Eclipse Ti2; Nikon, Tokyo, Japan). ImageJ software version 1.53j (https://imagej.nih.gov/ij/index.html) was used to generate the images.

### Biolistic delivery of plasmid DNA

The gold particles (0.6 µm diameter) used for biolistic delivery were washed with absolute ethanol, rinsed twice with sterilized water, and suspended in sterilized water to prepare a 30-mg/mL gold solution. The gold solution was dispensed (10 µL per shot) and mixed with 200–1000 ng of plasmid DNA(s) per shot in an agitating mixer, to which 4 µL of 0.1 M spermidine and 10 µL of 2.5 M calcium chloride per 10 µL of the gold solution were subsequently added. The resulting DNA-coated gold particles were collected by centrifugation at 3300 × *g* for 30 s. The DNA-coated gold particles were then washed once with 70% ethanol and twice with absolute ethanol and resuspended in absolute ethanol (10 µL per shot). Particle bombardment was performed using a PDS-1000/He system (Bio-Rad Laboratories, USA). The distance between the macrocarrier and target cells was adjusted to approximately 3.0 cm, the helium gas pressure was set to 1100 psi, and the degree of vacuum was set to at least − 25 inHg. For leaf bombardment, we used the leaves of mature *N. benthamiana* plants, which were taped to a plastic petri dish. The cut ends of the bombarded leaves were covered with a wet wipe and cultured at 25–30 °C for 20–24 h under humid and dark conditions. For pollen bombardment, pollen was collected immediately prior to bombardment and distributed on pollen germination medium solidified with 1% (w/v) NuSieve GTG Agarose (Lonza, Switzerland). Pollen germination media for *Nicotiana* (Wang and Jiang 2011) and torenia (Okuda et al. 2009) were used as previously described. The same pollen germination medium used for *Nicotiana* was used. After bombarding, the treated pollen was cultured directly on the medium and observed under an inverted fluorescence microscope (Eclipse Ti2, Nikon; AXIO imager A2, Zeiss). Biolistic delivery into leaves and pollen was examined twice, and gene introduction was evaluated based on the expression of fluorescent proteins.

### Detection of Cas9-induced genome editing

In addition to the CRISPR/Cas9 vector described above, plasmid vectors encoding fluorescent protein markers were simultaneously coated with gold particles. Bombarded leaves were observed under a fluorescence microscope (Eclipse Ti2, Nikon; AXIO imager A2, Zeiss) to confirm delivery. An area of approximately 1.5 cm diameter around the blast center was collected, immersed in a DNA extraction buffer [0.2 M Tris–HCl (pH 8.0), 0.25 M NaCl, 25 mM EDTA, and 0.5% (w/v) SDS], and frozen in liquid nitrogen prior to subsequent analysis. Genomic DNA was extracted from the leaf material using a homogenizer and sonicator. Pollen samples with or without fluorescent signals were collected together in the same DNA extraction buffer, and genomic DNA was extracted. For PCR analysis, the following steps were performed according to Nekrasov et al. (2013). Genomic DNA was digested with *Mly*I and PCR-amplified using the primer pair *PDS*_*Mly*IF and *PDS*_*Mly*IR (Nekrasov et al. 2013; Table S2). The PCR product thus obtained was subsequently used as a nested PCR template to remove non-specific DNA fragments. Because unedited wild-type fragments were detected in sequence analysis (described below), the first PCR product was digested with *Hin*fI before the nested PCR to reduce further amplification of the unedited wild-type fragments derived from undigested genome template with *Mly*I. The nested PCR product was cloned into the pCR-BluntII-TOPO vector (Thermo Fisher Scientific, USA) and introduced into *Escherichia coli* Mach1 T1^R^ competent cells (Thermo Fisher Scientific). To identify colonies possessing the fragment of the mutated *NbPDS3* gene, colony PCR was performed using primers M13 forward (M13f), M13 reverse (M13r), and *NbPDS3*_primer-m (Table S2). A pair of M13f and M13r primers amplifies approximately 664-bp fragments, which indicate insertion of the *NbPDS3*-derived sequence irrespective of mutations (Figure S1). When PCR product derived from the undigested genome was introduced into the vector as forward direction, 498 bp was amplified by primers M13r and NbPDS3_primer-m, whereas introduced into the vector as reverse direction, 478 bp was amplified by primers *NbPDS3*_primer-m and M13f (Figure S1). Accordingly, for colonies containing mutated *NbPDS3* fragments, weak or no bands were detected in 498 or 478 bp. A mutation in the *NbPDS3* gene was confirmed by sequence analysis of the plasmid vectors extracted from individual colonies.

### Aniline blue staining of pollinated pistils

Wild-type *N. benthamiana* and *N. tabacum* pistils were emasculated two days prior to pollination. The pollen spread on germination medium that had been hydrated for 15 min was used to pollinate the emasculated pistils using a dissecting needle. At 24 h after pollination, the pollinated flowers were collected, and the remaining petals and sepals were removed. The pistil was fixed with a 3:1 mixture of ethanol and acetic acid (v/v) overnight and treated with 1 N NaOH solution for 1 d. Thereafter, the pistils were stained with 0.1% (w/v) aniline blue in 0.1 M K_3_PO_4_ buffer for 1 d. Images were obtained using an Eclipse Ti2 fluorescence microscope under UV light. ImageJ software was used to generate the images.

### Semi-in vivo pollen tube growth assay using *N. tabacum*

*N. tabacum* pistils were emasculated two days prior to pollination. Plasmid vectors encoding *UBQ10p::sGFP* and *35Sp::H2B-tdTomato* were simultaneously coated on gold particles (mixture 1), as were the vectors encoding *LAT52p::mApple* and *UBQ10p::H2B-mClover* (mixture 2). Immediately prior to spreading onto the macrocarrier, mixtures 1 and 2 were mixed and then introduced into pollen via a single bombardment shot. Twenty-four hours after pollination, the pollinated style was excised with a razor 5 mm above the ovary and placed horizontally on the aforementioned pollen germination medium solidified with 1% (w/v) NuSieve GTG Agarose (Lonza), followed by incubation at 25–30 °C for 20 h under humid and dark conditions. Pollen tubes emerging from the cut end of the pistil were observed using an Eclipse Ti2 fluorescence microscope.

### In vivo experiments using *N. tabacum*

*N. tabacum* pistils were emasculated two days prior to pollination. Pollen was bombarded with mixture 1 and used immediately thereafter to pollinate the emasculated pistils. At 24 h post-pollination, the pollinated style was cut longitudinally using a razor, and at 24, 48, and 72 h post-pollination, the ovary wall was removed with forceps to facilitate observation of the pollen tube and ovule within the ovary. The samples were placed in 10% glycerol (v/v) and observed under an Eclipse Ti2 fluorescence microscope.

## Results

### The *AtUBQ10* promoter is suitable for driving transient gene expression in pollen germ cells

**Fig. 1:**
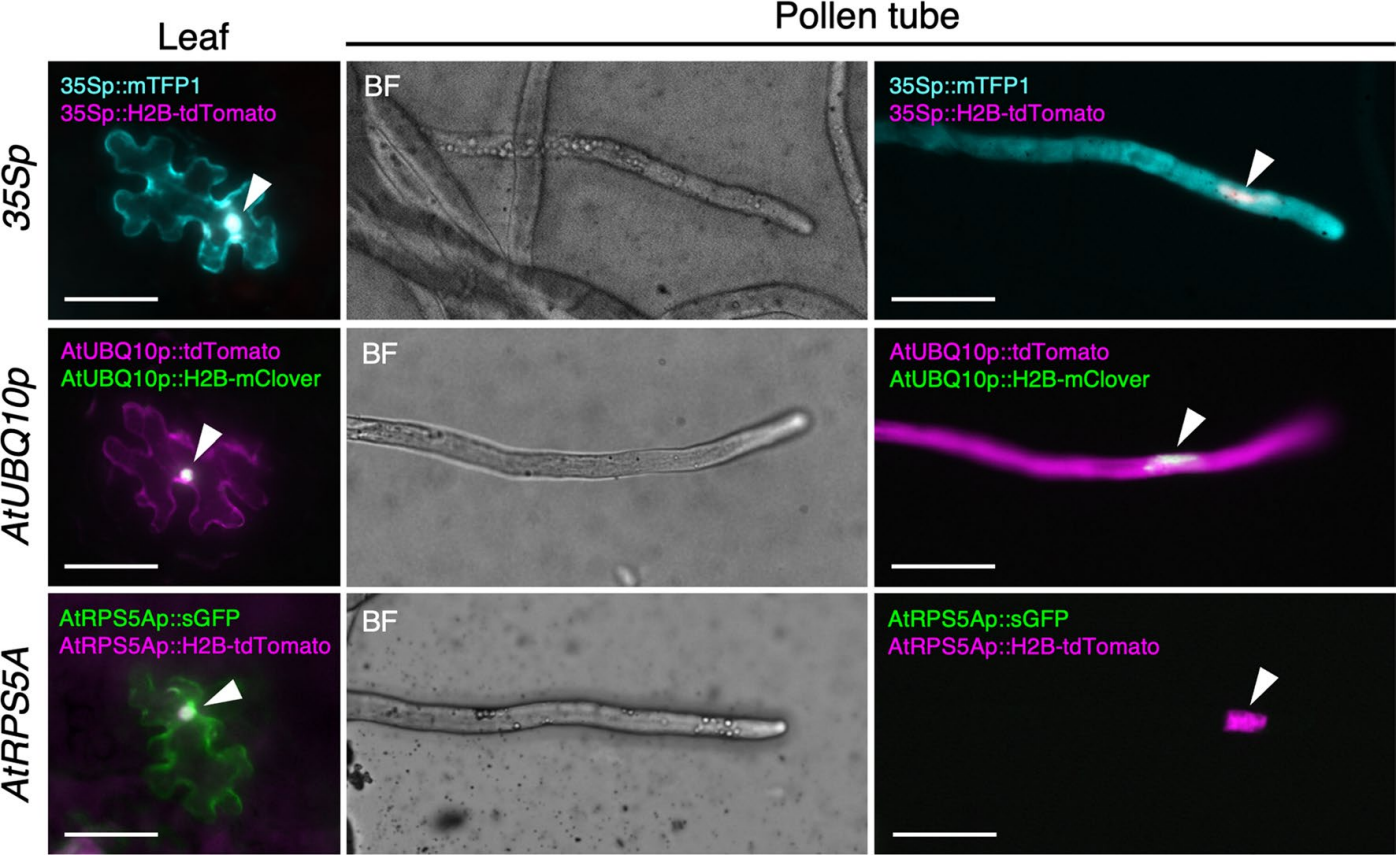
Promoter activity in *Nicotiana benthamiana*. CaMV 35S (35S), *Arabidopsis thaliana (At) UBQ10*, and *AtRPS5A* promoter activity in *N. benthamiana* leaves and pollen tubes. The epidermal cells in *N. benthamiana* leaves show the cytosolic and nuclear expression of fluorescent proteins driven by the 35S, *AtUBQ10*, or *AtRPS5A* promoter. Note that, as fluorescent proteins showing cytosolic expression were also localized in nuclei, pseudo-colors appear to be white in the nuclei (arrowheads). The *N. benthamiana* pollen tubes show the signals of fluorescent proteins driven by *35S* and *AtUBQ10* promoters in both cytosol and nucleus (arrowheads), whereas the signals of fluorescent proteins driven by the *AtRPS5A* promoter were observed only in the pollen tube nuclei in the merged fluorescent images. The plasmid DNAs used in the experiment are listed in Table S1. BF, bright field. Scale bars: 50 µm

To identify an effective promoter for driving the expression of Cas9 in pollen, we evaluated the activity of selected promoters in bombarded pollen based on fluorescent protein expression. Fluorescent proteins are driven under the control of cauliflower mosaic virus (CaMV) 35S, *A. thaliana* ribosomal protein S5A (*AtRPS5A*), and *A. thaliana* UBIQUITIN10 (*AtUBQ10*) promoters, which have previously been used as constitutive promoters (Liang et al. 2018; Nekrasov et al. 2013; Tsutsui and Higashiyama 2017), as well as the pollen vegetative cell-specific *LAT52* promoter derived from *S. lycopersicum* (Eady et al. 1995). The plasmid DNA vectors containing these promoters, which were introduced into the pollen and leaves, are summarized in Table S1. Tricellular pollen is generally short-lived relative to bicellular pollen, which increases the difficulty of handling this type of pollen (Hoekstra and Bruinsma 1975). In the present study, we used four species with bicellular pollen to investigate the introduction of the aforementioned plasmid DNA vectors, namely *N. benthamiana* (tobacco), *N. tabacum* (tobacco), *T. fournieri* (torenia), and *S. lycopersicum* (tomato), for which the in vitro induction of pollen tube germination has been established (Liang et al. 2018; Okuda et al. 2009; Paungfoo-Lonhienne et al. 2010; Zhang et al. 2020). Biolistic delivery into leaves and pollen was examined twice, and gene introduction was evaluated based on the expression of fluorescence proteins. Accordingly, we observed that in the bombarded leaves of all examined species, fluorescent proteins were expressed under the control of each of the three constitutive promoters (Fig. 1), whereas no fluorescent proteins were detected in leaves containing the pollen-specific *LAT52* promoter (Table 1). In the case of bombarded pollen, *AtUBQ10* and *AtRPS5A* promoters drove H2B-fused fluorescent protein expression in the nuclei of all examined species (Fig. 1). However, in the pollen cytoplasm, the *AtRPS5A* promoter did not drive the expression of fluorescent protein in *N. benthamiana* and *N. tabacum* (Table 1, Fig. 1). These differences in fluorescent protein expression were thought to be owing to differences in promoter activity among species. Similarly, the 35S promoter was active in the pollen of *N. benthamiana* and *N. tabacum*, but not in that of torenia or tomato (Table 1). A promoter that controls constitutive gene expression in generative cells is most suitable, as these cells contain the genome that is transmitted to the next generation. Accordingly, we used the *AtUBQ10* promoter to drive Cas9 in pollen transformed via particle bombardment. Furthermore, we used tobacco pollen in subsequent bombardment assays, as large amounts of pollen could be obtained from these plants.

**Table 1:** Transient expression of the fluorescent proteins by the biolistic delivery of plasmid DNA

		*35Sp* (cauliflower mosaic virus)	*AtRPS5Ap* (*A. thaliana*)	*AtUBQ10p* (*A. thaliana*)	*LAT52p* (*S. lycopersicum*)
*Pollen tube*					
Vegetative cell (cytoplasm)	*N. benthamiana*	+	−	+	+
	*N. tabacum*	+	−	+	+
	*T. fournieri*	−	+	+	+
	*S. lycopersicum*	−	+	+	+
Vegetative cell (nucleus)	*N. benthamiana*	+	+	+	N.D
	*N. tabacum*	+	+	+	N.D
	*T. fournieri*	−	+	+	N.D
	*S. lycopersicum*	−	+	+	N.D
Generative cell (nucleus)	*N. benthamiana*	+	+	+	N.D
	*N. tabacum*	+	+	+	N.D
*Leaf*					
Cytoplasm	*N. benthamiana*	+	+	+	−
	*N. tabacum*	+	+	+	−
	*T. fournieri*	+	+	+	−
	*S. lycopersicum*	+	+	+	−
Vegetative nucleus	*N. benthamiana*	+	+	+	N.D
	*N. tabacum*	+	+	+	N.D
	*T. fournieri*	+	+	+	N.D
	*S. lycopersicum*	+	+	+	N.D

### Efficiency of gene delivery into generative cells by particle bombardment

**Fig. 2:**
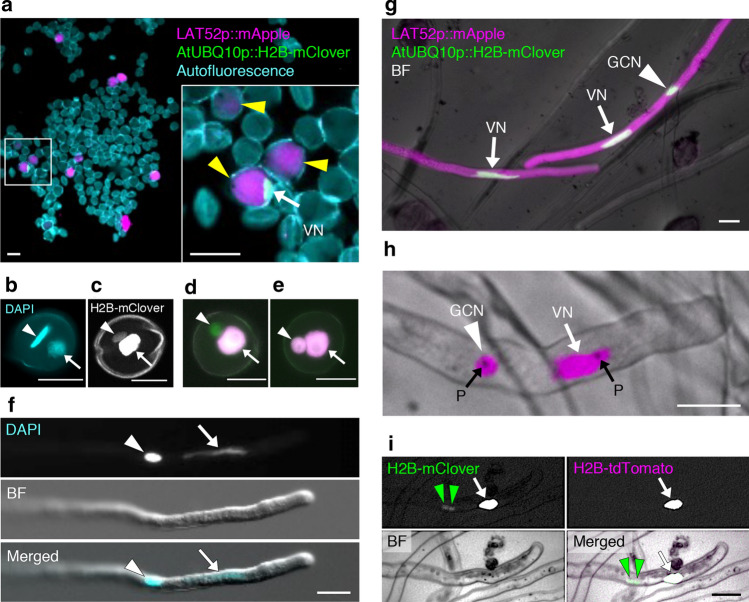
Biolistic delivery of plasmid DNAs into the vegetative and generative cells of *Nicotiana benthamiana* pollen and pollen tubes. **a** Bombarded pollen expressing fluorescent proteins at 6 h after bombardment. The inset shows a magnified view of the area demarcated by the white-bordered square. Yellow arrowheads indicate mApple-positive pollen cytosol (magenta; *LAT52p::mApple*), and the white arrow indicates a mClover-positive vegetative cell nucleus (green; *AtUBQ10p::H2B-mClover*). The outline of pollen grains is visualized by auto-fluorescence (cyan). **b** A DAPI-stained fixed pollen grain. **c:** A pollen of *AtUBQ10p::H2B-mClover* transgenic *N. benthamiana*. **d, e** Bombarded pollen of *AtUBQ10p::H2B-mClover* transgenic *N. benthamiana*. Green fluorescent signals of H2B-mClover indicate vegetative cell nucleus and generative cell nucleus in pollen, and red fluorescent signals of H2B-tdTomato indicate fluorescence derived from bombarded plasmid DNA (*35Sp::H2B-tdTomato*). When introduced into the pollen vegetative cells, only a vegetative nucleus was labeled (**d**), whereas when introduced into the generative cell, both nuclei were labeled (**e**). **f** A DAPI-stained fixed pollen tube. **g** Pollen tubes germinated from bombarded pollen at 20 h after bombardment. mApple and mClover fluorescence and bright-field (BF) images are merged. The arrows and arrowhead indicate mClover signals in vegetative nuclei and a generative cell nucleus, respectively. The right-hand pollen tube shows mClover signals in both vegetative and generative cell nuclei. **h** Pollen tubes germinated from pollen bombarded with *35Sp::H2B-tdTomato* plasmid DNA. RFP fluorescence and BF images are merged. An arrow and an arrowhead indicate H2B-tdTomato signals in a vegetative nucleus and a generative cell nucleus, respectively. Delivered dot-like gold particles are indicated by black arrows. **i** Germinated pollen tubes of *AtUBQ10p::H2B-mClover* transgenic *N. benthamiana*, containing two sperm cells at 10 h after bombardment with *35Sp::H2B-tdTomato* plasmid DNA. Green arrowheads indicate two sperm cell nuclei and arrows indicate a vegetative nucleus. A green signal was detected in the nuclei of both vegetative and sperm cells, whereas a magenta signal was observed only in the vegetative cell. The white arrows and white arrowheads indicate the vegetative and generative cell nucleus, respectively. VN, vegetative nucleus; GCN, generative cell nucleus; P, gold particles within the generative cell and pollen tube. Scale bars: **a** 50 µm, **b–i** 20 µm

In general, the efficiency with which genes are delivered by particle bombardment is typically as low as several percent (Wang and Jiang 2011). Given that genome editing occurs in only a fraction of the bombarded pollen, we initially investigated the efficiency with which genes were delivered into pollen. Plasmids carrying fluorescent protein-coding sequences driven under the control of the aforementioned promoters were introduced into the pollen of *N. benthamiana* by particle bombardment. We simultaneously introduced two types of plasmid DNA encoding mApple and H2B-mClover into the same pollen; accordingly, we observed mApple signals in the cytoplasm, whereas mClover signals were localized in the nuclei, indicating that subcellular structures in pollen and pollen tubes were labeled transiently (Fig. 2a). Staining of the nuclei of the wild-type pollen indicated a spindle-shaped generative cell (arrowhead in Fig. 2b) and vegetative nucleus (arrow in Fig. 2b). We also produced *Agrobacterium*-mediated transgenic *N. benthamiana* expressing *H2B-mClover* under the control of the *AtUBQ10* promoter and detected the corresponding H2B-mClover signals in the nuclei of both vegetative and generative cells (Fig. 2c). When plasmid DNAs were introduced into pollen vegetative cells, only the vegetative nucleus was labeled (Fig. 2d), whereas when they were also introduced into generative cells, both nuclei were labeled (Fig. 2e). After pollen germination, the vegetative cell nucleus was localized in the tip, and the generative cell nucleus was located in the stained pollen tube (Fig. 2f). Additionally, the observed expression of delivered genes in the vegetative and generative cells of the pollen tubes indicated that the gold particles had penetrated the generative cell enclosed within the pollen (Fig. 2g). Interestingly, in some pollen tubes, we observed dot-like structures, which were assumed to be 0.6-μm gold particles (Fig. 2h). Furthermore, following bombardment, some pollen tubes were found to contain two sperm cells that were divided from the generative cell (Fig. 2i). Although nuclear signals of both vegetative and sperm cells were observed, fluorescence signals derived from the delivered plasmid DNA were detected only in the vegetative cells, indicating that gold particles were introduced only into the vegetative cells of pollen and that generative cell division occurred to produce two sperm cells.

To obtain an estimate of the efficiency with which genes are delivered into the vegetative and generative cells of pollen, we subsequently determined the ratio of transformed pollen grains to total pollen grains. The estimated percentages of the vegetative cells of transformed pollen showing expression of mApple in the cytoplasm and mClover in vegetative nuclei were 1.2% (18.3 ± 6.5 pollen grains) and 1.1% (16.0 ± 4.7 pollen grains), respectively (1479 ± 298.5 pollen grains per experiment, *n* = 3). This indicated that there was little difference in the efficiency of gene delivery and expression regardless of protein localization (i.e., cytoplasm or nucleus). However, we estimated that only 0.2% of the transformed pollen showed mClover signals in generative cell nuclei, and in such pollen, both vegetative and generative nuclei were labeled (3.5 ± 1.0 pollen grains). This indicates that approximately one-sixth of the delivered pollen was also introduced into the generative cells. Based on these observations, we concluded that particle bombardment is applicable for gene delivery into vegetative and generative cells.

### Genome editing in pollen can be induced by exogenous CRISPR/Cas9 components

**Fig. 3:**
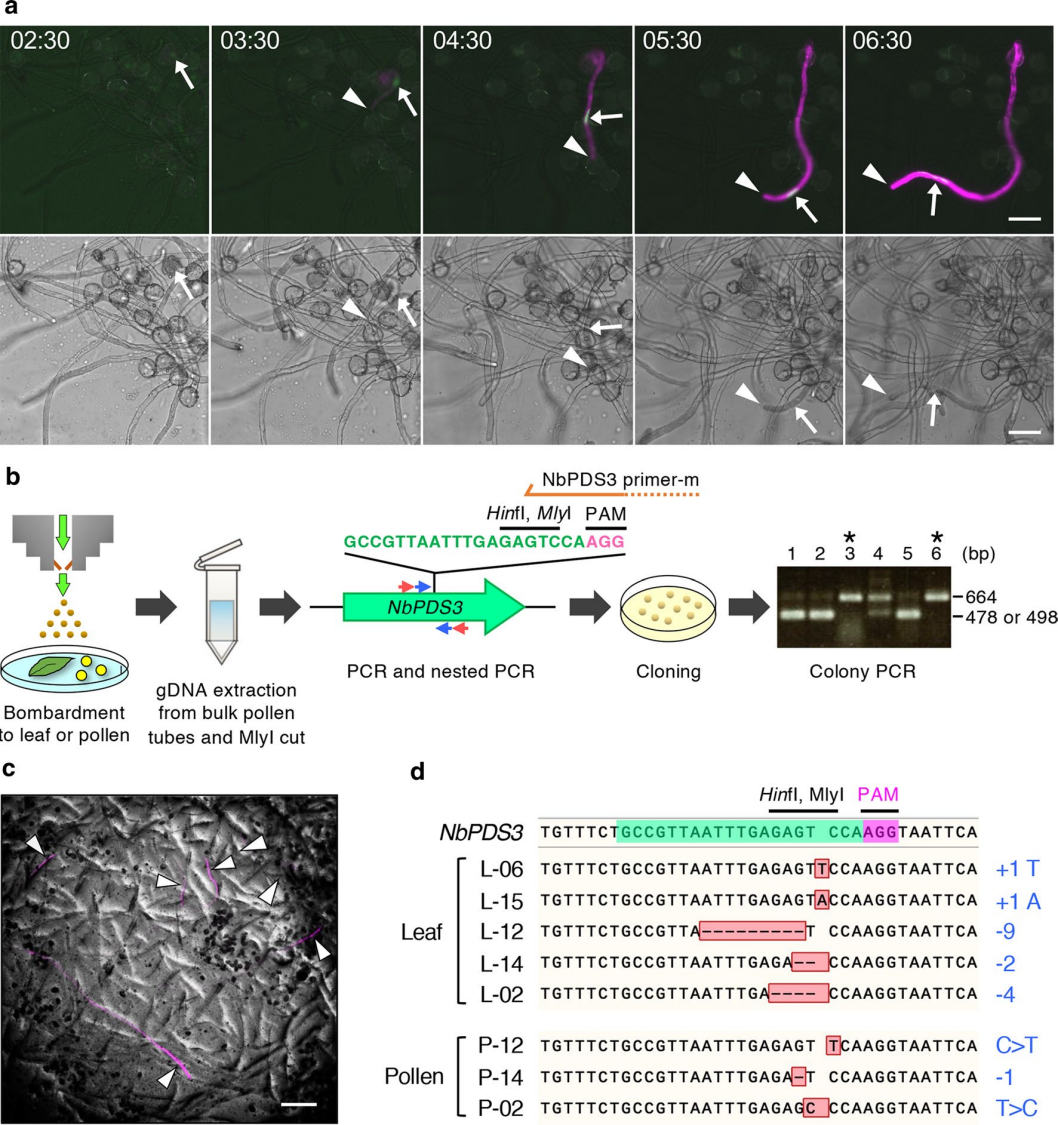
Cas9-induced genome editing via biolistic delivery of CRISPR/Cas9 plasmid DNA introduced into *Nicotiana benthamiana* leaves and pollen. **a** Time-lapse images of fluorescent protein expression in bombarded pollen. Fluorescent (upper panels) and bright-field (BF) (lower panels) images are shown. The signals of tdTomato and H2B-mClover proteins driven by the *AtUBQ10* promoter are shown as magenta and green, respectively. The times denote the period elapsed post-bombardment (hours:minutes). The arrows and arrowheads indicate mClover in the nucleus and the tip of pollen tube of the bombarded pollen. See also Video S1. **b** Schematic representation of the experimental procedure used to identify mutations in the leaves or pollen into which CRISPR/Cas9 plasmid DNA has been introduced. The bombarded leaves or bulk pollen tubes were transferred to a microtube containing DNA extraction buffer, and genomic DNA was then extracted. The genomic DNA was digested with *Mly*I, and a part of the *N. benthamiana PDS3* (*NbPDS3*) gene was PCR-amplified using a primer pair represented by red arrows. Nested PCR was performed directly or after *Hin*fI digestion using a primer pair represented by blue arrows. The partial sequence of the *NbPDS3* gene contains an sgRNA-targeting sequence (green). The PAM sequence is shown in magenta. The nested PCR products were cloned into a cloning vector and transformed into *Escherichia coli*. Colony PCR was performed using three primer sequences; *NbPDS3* primer-m, of which the 3ʹ end was matched to the wild-type *NbPDS3* sequence (orange arrow), and M13 forward and M13 reverse on the vector. In the agarose gel image showing PCR bands, 664 bp represents PCR products of M13 forward/reverse. Another band at 478 or 498 bp shows PCR products of M13 forward or reverse/*NbPDS3* primer-m. When a mutation on the *Hin*fI-*Mly*I site was generated by CRISPR–Cas9, the lower band (478 or 498 bp) was not detected. The samples shown in lanes 3 and 6 are mutated. See also Figure S1. **c** Pollen tubes derived from bombarded pollen expressing tdTomato at 18 h after bombardment (arrowheads). Genomic DNA was extracted from bulk pollen tubes. **d** Sequence analysis of the mutation in bombarded leaves and pollen. The upper sequence is the wild-type *NbPDS3* gene. The sequences amplified from both bombarded leaf and pollen containing the CRISPR/Cas9 vector show mutations in the *NbPDS3* gene. The changes in length and sequence are shown to the right. Scale bars: **a** 50 µm, **c** 200 µm

To facilitate CRISPR–Cas9-mediated genome editing, the introduced gene must initially be transcribed and translated, which results in a time lag before the effects can be detected. Therefore, we performed time-lapse imaging of the bombarded pollen to estimate the length of time required prior to the detection of transient expression of introduced DNA in vitro. We found that fluorescent signals of mClover encoded by the *AtUBQ10p::H2B-mClover* plasmid (sSNv28; Table S1) appeared at 2.5–3.5 h after bombardment, whereas those of tdTomato encoded by the *AtUBQ10p::tdTomato* plasmid (sSNv26; Table S1) appeared at 3.5–4.5 h (Fig. 3a, Video S1). In this regard, it has been established that pollen tube germination in *N. benthamiana* commences after 0.5–5 h on agarose media and thereafter grows for approximately 24 h (Paungfoo-Lonhienne et al. 2010). Accordingly, we decided to analyze CRISPR/Cas9 plasmid-mediated genome editing in *N. benthamiana* pollen 20–24 h post-bombardment. To investigate whether the biolistically delivered CRISPR/Cas9 system is functional in pollen, we introduced a plasmid DNA consisting of a human codon-optimized Cas9 gene and an *A. thaliana U6.26* promoter (*AtU6.26*)-driven sgRNA cassette (Tsutsui and Higashiyama 2017). The functioning of the *AtU6.26* promoter and sgRNA in *N. benthamiana* leaves was confirmed by transient expression via agro-infiltration (Nekrasov et al. 2013). We initially introduced the *AtUBQ10p::Cas9/U6.26p::NbPDS3-sgRNA* plasmid (sSNv21; Table S1) into *N. benthamiana* leaves by particle bombardment and then identified different mutation patterns based on sequence analysis (Fig. 3b–d and Fig. S1). Analysis of the sequences obtained from 116 clones derived from the PCR product shown in lanes 3 and 6 of the gel depicted in Fig. 3b revealed the presence of indels in 17 of these clones. These mutations could be grouped into five different types: 9-, 4-, and 2-bp deletions or 1-bp insertions of either T or A (Fig. 3d). The same plasmid was subsequently delivered into *N. benthamiana* pollen. Bulk pollen tubes with and without fluorescent signals (Fig. 3c) were collected, and genomic DNA was extracted. Sequence analysis of 168 clones derived from the PCR product revealed the presence of deletions or substitutions in 33 of these clones, which could be grouped into three different types: 1-bp deletion or substitutions of either C to T or T to C (Fig. 3d). No mutations were detected in genomic DNA extracted from pollen tubes bombarded without plasmid DNA. These results indicate that the transient expression of plasmid DNA, including CRISPR/Cas9, delivered via particle bombardment, induces genome editing in the leaves and pollen of *N. benthamiana*.

### Visualization of fertilization process of bombarded pollen in vivo

**Fig. 4:**
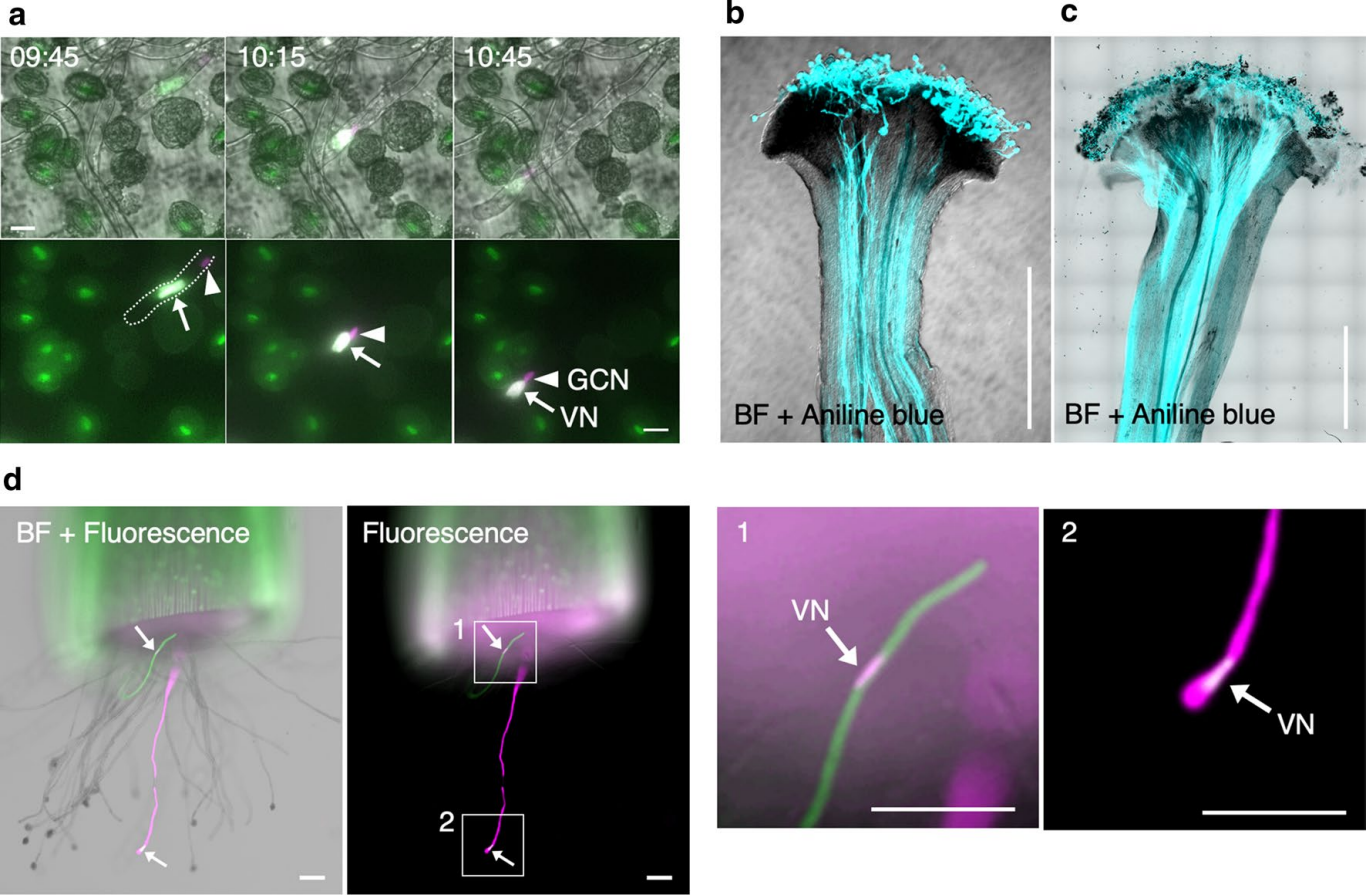
Pollen tubes derived from pollen bombarded in vitro and semi-in vivo experiments. **a** Delivery of the bombarded generative cell in a pollen tube of a transgenic *Nicotiana benthamiana* plant. The vector *35Sp::H2B-tdTomato* was introduced into pollen from *AtUBQ10p::H2B-mClover*. Note that the generative cell nucleus appears magenta in color, whereas the vegetative nucleus appears white, due to the lower level of the background expression of *H2B-mClover* in the GCN than VN of the transgenic line, as shown in Fig. 2d, f. Upper panels are merged images of bright-field (BF), GFP, and RFP images, and lower panels are merged images of GFP and RFP images. The dashed line in the first image of the lower panels denotes the outline of the pollen tube, and the time indicates the period elapsed post-bombardment (hours:minutes). Arrows and arrowheads indicate the vegetative and generative cell nuclei, respectively. See also Video S2. **b, c** Pollen tube germination on pistils using pollen hydrated on agarose germination medium. After spreading on the agarose medium, pollen grains were collected and used to pollinate the emasculated stigmas of *N. benthamiana* (**b**) and *N. tabacum* (**c**) pistils. Pollinated pistils were collected 24 h after pollination and stained with aniline blue solution. BF and ultraviolet (UV)-illuminated images were merged. **d** Semi-in vivo analysis of pollen tubes in *N. tabacum* pollinated after bombardment. Among the pollen tubes that emerged from the cut end of a pollinated pistil, two fluorescent-positive pollen tubes were observed. Arrows indicate fluorescent signals in the vegetative nuclei. Magnified images are also shown. VN, vegetative nucleus; GCN, generative cell nucleus. Scale bars: **a** 20 µm, **b**, **c** 500 µm, **d** 100 µm

**Fig. 5:**
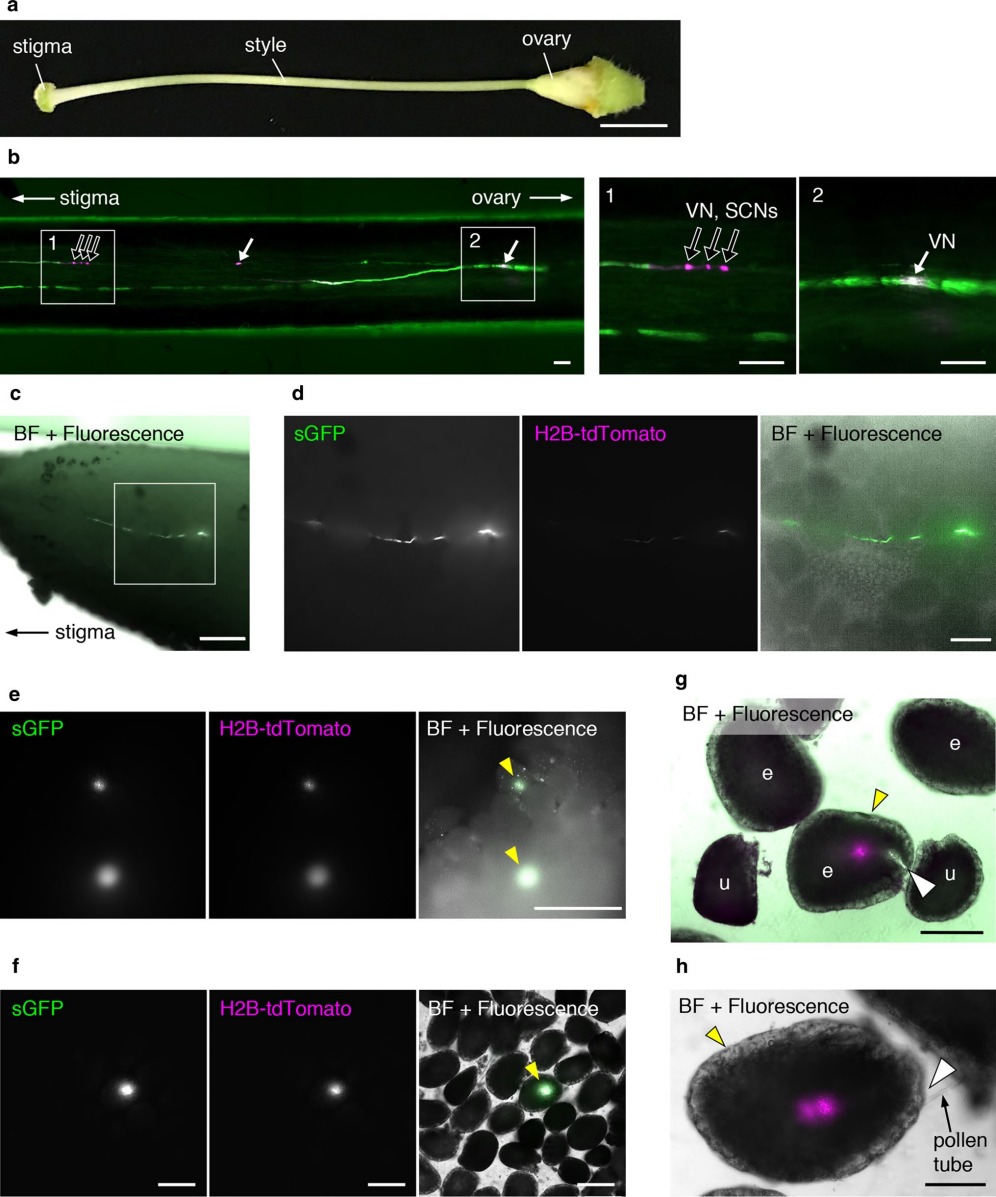
In vivo pollination of bombarded pollen with *AtUBQ10p::sGFP* and *35Sp::H2B-tdTomato* plasmid DNAs in *N. tabacum*. **a** The pistil of *N. tabacum*. **b** Vertical section of a style with bombarded pollen 24 h after pollination. Pollinated style was hand dissected by tweezers and placed on the 10% glycerol. Magnified images are shown in the right panel. **c** Pollen tubes from the bombarded pollen on the placenta 48 h after pollination. Ovary wall was removed by tweezers and placed on the 10% glycerol. Magnified images are shown in **d**. **e** Two ovules on the placenta that received pollen tube from the bombarded pollen 48 h after pollination. Ovary wall was removed by tweezers and placed on 10% glycerol. **f** Dissected ovules on 10% glycerol from the placenta 48 h after pollination with bombarded pollen. Single ovule that receives pollen tube from the bombarded pollen. **g, h** Dissected ovules on 10% glycerol from the placenta 72 h after pollination with bombarded pollen. Enlarged ovules that receive pollen tube from the bombarded pollen show fluorescent signals. White arrows indicate the vegetative nucleus; black arrows show vegetative and generative nuclei. Yellow arrowheads indicate the ovule showing fluorescent signal derived from the bombarded pollen. White arrowheads indicate the micropyle of the ovule showing fluorescent signal derived from the bombarded pollen. VN, vegetative nucleus; GCN, generative cell nucleus; SCN, sperm cell nucleus; e, enlarged ovule; u, unfertilized ovule. Scale bars: **a, c, e** 500 µm, **b, f, h** 100 µm, **d, g** 200 µm

To investigate whether bombarded pollen retains fertilization capacity in vivo, we used a nuclear-labeled marker line to investigate pollen tube germination and the delivery of generative cells. For bombardment, we used pollen derived from a *UBQ10p::H2B-mClover* transformant into which plasmid DNA containing the *UBQ10p:H2B-tdTomato* sequence (DKv277; Table S1) was introduced. Thereafter, we performed time-lapse imaging of pollen tube elongation for 18 h after bombardment. Time-lapse observation revealed the delivery of bombarded generative cells within the elongating pollen tube (Fig. 4a, Video S2). In some species, pollen is unable to germinate on the stigma when the pollen has initially been hydrated on the medium (Zuberi and Dickinson 1985). Thus, we investigated whether *N. benthamiana* and *N. tabacum* pollen that had been hydrated on the germination medium could germinate on the pistil. Following hydration, pollen was immediately used to pollinate the emasculated stigmas within 15 min. The pollinated pistils were then fixed and stained with aniline blue solution, and observations indicated that the hydrated pollen of both *N. benthamiana* and *N. tabacum* germinates on the respective pistils in vivo (Fig. 4b, c). To enable direct observations of bombarded pollen tube elongation, we conducted a semi-in vivo pollen tube growth assay (Palanivelu and Preuss 2006). Owing to the thin style, the pistils of *N. benthamiana* are difficult to dissect without causing damage; therefore, we used *N. tabacum*, which has thick hard pistils, for this assay. Wild-type pollen bombarded with a mixture of plasmid DNAs encoding fluorescent proteins driven by *AtUBQ10* or 35S promoters was immediately used to pollinate emasculated pistils, and pollen tubes were observed, including those derived from bombarded pollen, emerging from the end of the cut style (Fig. 4d). Moreover, we found that the vegetative nucleus in the pollen tube was labeled and that the lengths of the emerging pollen tubes derived from bombarded pollen were similar to those of other pollen tubes that had germinated from non-bombarded pollen. Furthermore, the fertilization process of the bombarded pollen after pollen tube germination was observed in vivo (Fig. 5). The emasculated wild-type *N. tabacum* pistil was pollinated with bombarded pollen with plasmid DNA, including *AtUBQ10p::sGFP* and *35Sp::H2B-tdTomato* sequences. Dissection of pistils at 24 h after pollination (Fig. 5a) revealed that the pollen tubes derived from bombarded pollen had elongated within the longitudinal section of the style in vivo (Fig. 5b). Moreover, we observed that some pollen tubes underwent cell division, resulting in the production of two sperm cells and a single vegetative cell within the pollen tube (black arrows in Fig. 5b). Additionally, the pistils were dissected 48 h after pollination. We observed that bombarded pollen elongated on the placenta in the ovary (Fig. 5c, d) and that some ovules had received pollen tubes derived from bombarded pollen grains 48 h after pollination (Fig. 5e). When bombardment was performed twice and two flowers were pollinated each, one, two, three, and five ovules showed fluorescence signals in each ovary at 48 h after pollination. Fluorescence signals derived from pollen tube cytoplasm reporters were detected as a large spot of fluorescence signals inside the ovules (Fig. 5f). Furthermore, some of the enlarged ovules that seemed to be fertilized remained fluorescent 72 h after pollination. The fluorescence of the cylindrical green sGFP and spot-shaped red H2B-tdTomato signals was observed inside an enlarged ovule (Fig. 5g). Interestingly, two red H2B-tdTomato fluorescence signals were occasionally observed inside ovules 72 h after pollination, which were suggested as the multiple nuclei derived from bombarded pollen, embryo, or endosperm (Fig. 5h). This indicated that the pollen tubes released their contents inside the ovule, which were detected as a convenient marker of targeted ovules (Palanivelu and Preuss 2006). These observations indicate that even in vivo, bombarded pollen germinates and gives rise to pollen tubes that undergo subsequent elongation and deliver their contents, including sperm cells, into the ovule.

## Discussion

Animal germ cells, such as sperm and eggs, contain a genome that develops into individuals of the next generation. To obtain gene-modified organisms by inducing heritable genetic changes, genome editing has been studied using germline cells, zygotes, and embryos in animals (Cooper et al. 2017; Koslova et al. 2020; Lea and Niakan 2019). With respect to plants, somatic cells are generally used for genome engineering as they retain totipotency (Ikeuchi et al. 2015). Recently, rice zygote has also been used to produce genome-modified plants (Toda et al. 2019). However, sophisticated anatomical techniques are required to isolate zygotes, since they are embedded deep within tissues (Mizuta and Higashiyama 2018). Moreover, tissue culture is necessary to generate genome-edited plants after zygote delivery. Compared with zygotes and female germ cells, male gametophyte pollen is readily isolated and has a simple structure comprising two sperm cells. Pollen can thus be used as a “vector” for gene engineering of plant germ cells via fertilization (Resch and Touraev 2010). With regard to the number of cells contained within pollen grains, angiosperm pollen is generally divided into two types: bicellular and tricellular (Russell and Jones 2015). Approximately 30% of angiosperms produce tricellular pollen comprising two sperm cells at anthesis (Brewbaker 1967). The other 70% of angiosperms, including *N. benthamiana* and *N. tabacum*, produce bicellular pollen grains that contain a generative cell as a precursor of sperm cells. After anthesis, the generative cell undergoes mitosis to form two sperm cells within the pollen tube (Hackenberg and Twell 2019). These two sperm cells fertilize an egg cell and a central cell, respectively (Berger et al. 2008). Consequently, only genetic material in the sperm cell that fertilizes the egg cell is transmitted to the progeny. In the present study, we detected the expression of delivered fluorescent proteins 2–3 h post-bombardment (Fig. 3a and Video S1) and division into two sperm cells was observed at approximately 10 h (Fig. 2i), which is comparable to observations of pollen tubes derived from non-bombarded wild-type pollen (Tian et al. 2005). Expression of the Cas9 protein is typically detectable within 5 h after transfection (Fajrial et al. 2020), and the findings of the present study indicate that when generative cells are mutated by biolistic delivery of CRISPR/Cas9, it is possible that the mutation is inherited in both sperm cells in bicellular pollen. Bicellular pollen is considered to be more suitable than tricellular pollen for the genome engineering of sperm cells in pollen tubes. In general, the DNA repair pathway is closely associated with the cell cycle, in which it plays a key role in maintaining genomic integrity during mitosis (Mao et al. 2008). The cell cycle of pollen vegetative cells enters the G_0_ phase, whereas at anthesis, the cell cycle of the generative cell is in the G_1_ S or G_2_ phase, depending on the species (Borg and Twell 2010; Friedman 1999). Our observations in the present study indicate that the efficiency of genome editing in the pollen tube is lower than that in leaves, which could be attributed to the fact that the cells undergo only a single round of mitosis or that the cell cycle of the generative cell is at the late interphase. Moreover, this may be due to chromatin condensation in generative cells.

The initial step in plant genetic engineering is the delivery of genes into plant cells. However, the protective rigid cell walls of most angiosperms tend to limit the effective delivery of most molecular types. Conventional methods used to deliver genes into plant cells can be grouped into three categories: physical, chemical, or biological (Birch 1997; Han and Kim 2019). Among these, one of the most commonly used biological approaches for generating gene-modified plants is *Agrobacterium*-mediated transformation (Bevan 1984). However, for many species, including those of economic importance, a drawback of this technique is the prerequisite of tissue culturing steps to facilitate plant regeneration. As an alternative, a physical gene delivery approach, biolistic particle delivery (also referred to as particle bombardment or gene gun delivery), has been widely used in plant species, including non-model plants (Sachin Rustgi 2020). This method, which is effective regardless of species or tissue type, entails a simple and rapid procedure that can efficiently deliver a range of molecular types, including DNA, RNA, proteins, and dyes (Martin-Ortigosa and Wang 2014; Wang and Jiang 2011; Zhang et al. 2016). In this regard, when seeking to introduce DNA, such as plasmid vectors, a promoter that functions constitutively in the introduced cells is required. For example, although the *AtRPS5A* promoter has been shown to be efficient for driving the expression of Cas9 in *A. thaliana* germ cells via *Agrobacterium*-mediated transformation (Ordon et al. 2020; Tsutsui and Higashiyama 2017), we found that it tends not to function constitutively when delivered via particle bombardment in both *N. benthamiana* and *N. tabacum* pollen tubes (Table 1 and Fig. 1). In contrast, we observed that the *AtUBQ10* promoter showed high constitutive activity in the pollen tubes of the four species examined in the present study (Table 1). Under transient conditions, the CaMV 35S promoter showed activity in tobacco pollen, but not in either torenia or tomato pollen (Table 1). Using transformants, the CaMV 35S promoter is not active in *Arabidopsis* pollen, whereas it is active in tobacco pollen (Wilkinson et al. 1997). The CaMV 35S promoter activity in pollen may differ among species. It is thus conceivable that the *AtUBQ10* promoter sequence contains a universal sequence that enhances the efficiency of genome editing in pollen tubes (Zheng et al. 2020).

Although biolistic delivery is typically used to facilitate transient expression, methods for producing transformants or genome-edited plants from bombarded cells have also been reported. Recent studies have reported successful editing of plant genetic material via particle bombardment delivery of plasmids, in vitro transcripts, or ribonucleoprotein complexes (RNPs) of CRISPR/Cas9 complexes using wheat and maize embryo-derived calli (Liang et al. 2018; Svitashev et al. 2016, 2015). Furthermore, an *in planta* transformation method using biolistic delivery has been reported that does not require callus culture and regeneration (Hamada et al. 2017). In the case of pollen, transformants produced via regeneration from bombarded pollen have also been reported (Stöger et al. 1995). In tobacco, only five antibiotic-resistant seeds harboring transgenes were identified from 30,000 seeds (Touraev et al. 1997). In the present study, we succeeded in introducing genes into pollen via particle bombardment, and the efficiency achieved was typically low. We detected a total of 11 ovules showing fluorescent expression in four ovaries pollinated with bombarded pollen (Fig. 5e, f). Given that the number of seeds in *N. tabacum* is approximately 530–1000 per ovary (Touraev et al. 1997), it is estimated that the percentage of fluorescent-expressed ovules is 0.275–0.519% of the total. One-sixth of the pollen was delivered into the generative cells, and it is estimated that 0.046–0.086% of the total ovules are derived from bombarded pollen introduced into the generative cell. This suggests that approximately 1 in 2000 seeds is likely to contain a gene-edited embryo. However, genome editing occurs only in a fraction of them; thus, it is expected that the number of seeds containing edited genomes will be extremely small. Compared with other gene delivery methods, such as *Agrobacterium*-mediated methods, such low efficiency is an issue in some plants with few seeds. Therefore, for this method to be widely used in plants, it is essential that it is able to efficiently detect genetically modified seeds among a large excess of unmodified seeds. Additionally, efficient production of genome-edited plants will require efficient biolistic delivery and improved efficacy of genome editing. With regard to the former, our detection method of biolistic delivery in pollen and pollinated pistils would contribute to the assessment of efficiency at various reproductive stages. In the latter regard, a number of approaches aimed at enhancing the efficiency of genome editing have been reported, including the modification of Cas9 (Ling et al. 2020; Osakabe et al. 2020), design of guide RNA (Moon et al. 2019), use of RNPs (Liang et al. 2018; Svitashev et al. 2016), and use of small chemical compounds (Yu et al. 2015). Various selection methods have also been reported, including those based on antibiotic resistance (Chesnokov and Manteuffel 2000) and herbicide resistance, by targeting endogenous genes (Han and Kim 2019). When genome editing occurs in pollen and the plasmid DNA is not delivered to the egg, the target locus in the resulting seed is expected to be heterozygous. In *N. tabacum*, zygote formation occurs 72–96 h after pollination (Zhao et al. 2020). Interestingly, the fluorescence signal derived from the bombarded pollen remained inside the enlarged ovule until 72 h after pollination (Fig. 5g, h). This suggests that the exogenous gene products derived from the bombarded pollen remain until the zygotic stage, which may lead to homozygous mutations. To discuss this possibility, more efficient biolistic delivery and seed detection methods are needed. These findings and our detection method will contribute to further advances in the engineering of plant genomes, including those of economically important crops and non-model plants.

## Supplementary Information

**Table S1** List of the plasmid DNA vectors used in this study.

**Table S2** List of the primers used in this study. Figure S1 Schematic representation of the experimental procedure used to identify mutations in the leaves or pollen into which CRISPR/Cas9 plasmid DNA has been introduced. The genomic DNA was digested with MlyI and was used to PCR using a primer pair PDS_MlyIF/PDS_MlyIR represented by red arrows. Nested PCR was performed directly or after HinfI digestion using a primer pair NbPDS3_nest_F/NbPDS3_nest_R represented by blue arrows. The nested PCR products were cloned into a cloning vector and transformed into Escherichia coli. Colony PCR was performed using three primer sequences; NbPDS3_primer-m, of which the 3ʹ end was matched to the wild-type NbPDS3 sequence (orange arrow), and M13 forward and M13 reverse on the vector. In the agarose gel image showing PCR bands, 664 bp represents PCR products of M13 forward/reverse. Another band at 498 bp shows PCR products of M13r/ NbPDS3_primer-m, and 478 bp shows PCR products of NbPDS3_primer-m/M13f. When a mutation on the HinfI–MlyI site was generated by CRISPR–Cas9, the lower band (478 or 498 bp) was not detected. The sgRNA-targeting sequence in the NbPDS3 gene is shown as green box. The PAM sequence is shown as magenta box. Figure S1 Schematic representation of the experimental procedure used to identify mutations in the leaves or pollen into which CRISPR/Cas9 plasmid DNA has been introduced. The genomic DNA was digested with MlyI and was used to PCR using a primer pair PDS_MlyIF/PDS_MlyIR represented by red arrows. Nested PCR was performed directly or after HinfI digestion using a primer pair NbPDS3_nest_F/NbPDS3_nest_R represented by blue arrows. The nested PCR products were cloned into a cloning vector and transformed into Escherichia coli. Colony PCR was performed using three primer sequences; NbPDS3_primer-m, of which the 3ʹ end was matched to the wild-type NbPDS3 sequence (orange arrow), and M13 forward and M13 reverse on the vector. In the agarose gel image showing PCR bands, 664 bp represents PCR products of M13 forward/reverse. Another band at 498 bp shows PCR products of M13r/ NbPDS3_primer-m and 478 bp shows PCR products of NbPDS3_primer-m/M13f. When a mutation on the HinfI–MlyI site was generated by CRISPR–Cas9, the lower band (478 or 498 bp) was not detected. The sgRNA-targeting sequence in the NbPDS3 gene is shown as green box. The PAM sequence is shown as magenta box. Figure S1 Schematic representation of the experimental procedure used to identify mutations in the leaves or pollen into which CRISPR/Cas9 plasmid DNA has been introduced. The genomic DNA was digested with MlyI and was used to PCR using a primer pair PDS_MlyIF/PDS_MlyIR represented by red arrows. Nested PCR was performed directly or after HinfI digestion using a primer pair NbPDS3_nest_F/NbPDS3_nest_R represented by blue arrows. The nested PCR products were cloned into a cloning vector and transformed into Escherichia coli. Colony PCR was performed using three primer sequences; NbPDS3_primer-m, of which the 3ʹ end was matched to the wild-type NbPDS3 sequence (orange arrow), and M13 forward and M13 reverse on the vector. In the agarose gel image showing PCR bands, 664 bp represents PCR products of M13 forward/reverse. Another band at 498 bp shows PCR products of M13r/ NbPDS3_primer-m and 478 bp shows PCR products of NbPDS3_primer-m/M13f. When a mutation on the HinfI–MlyI site was generated by CRISPR–Cas9, the lower band (478 or 498 bp) was not detected. The sgRNA-targeting sequence in the NbPDS3 gene is shown as green box. The PAM sequence is shown as magenta box.

**Figure S1** Schematic representation of the experimental procedure used to identify mutations in the leaves or pollen into which CRISPR/Cas9 plasmid DNA has been introduced. The genomic DNA was digested with *Mly*I and was used to PCR using a primer pair *PDS*_MlyIF/*PDS*_MlyIR represented by red arrows. Nested PCR was performed directly or after *Hin*fI digestion using a primer pair *NbPDS3*_nest_F/*NbPDS3*_nest_R represented by blue arrows. The nested PCR products were cloned into a cloning vector and transformed into *Escherichia coli*. Colony PCR was performed using three primer sequences; *NbPDS3*_primer-m, of which the 3ʹ end was matched to the wild-type *NbPDS3* sequence (orange arrow) and M13 forward and M13 reverse on the vector. In the agarose gel image showing PCR bands, 664 bp represents PCR products of M13 forward/reverse. Another band at 498 bp shows PCR products of M13r/ *NbPDS3*_primer-m and 478 bp shows PCR products of *NbPDS3*_primer-m/M13f. When a mutation on the *Hin*fI-*Mly*I site was generated by CRISPR–Cas9, the lower band (478 or 498 bp) was not detected. The sgRNA-targeting sequence in the *NbPDS3* gene is shown as green box. The PAM sequence is shown as magenta box.

**Video S1** Time-lapse images of fluorescent protein expression in the bombarded pollen. See also Fig. 3a. The times denote the period elapsed after bombardment (hours:minutes). Arrows and arrowheads indicate mClover in the nucleus and the tip of the pollen tube of the bombarded pollen, respectively.

**Video S2** Movement of the generative cell nucleus in a pollen tube germinated from bombarded pollen. See also Fig. 4a. The times denote the period elapsed after bombardment (hours:minutes). Arrows and arrowheads indicate the vegetative and generative cell nuclei, respectively.

Below is the link to the electronic supplementary material.

Supplementary file1 (PDF 59 kb)

Supplementary file2 (PDF 39 kb)

Supplementary file3 (TIF 393 kb)

Supplementary file4 (MOV 373 kb)

Supplementary file5 (MOV 508 kb)

## Data Availability

The authors confirm that the data supporting the findings of this study are available within the article and its supplementary materials.
