# Supplementary material for: Detection of a biolistic delivery of fluorescent markers and CRISPR/Cas9 to the pollen tube
Source: Plant Reprod. 2021 Jun 19;34(3):191–205. doi: 10.1007/s00497-021-00418-z (PMC8360903; doi:10.1007/s00497-021-00418-z)
Supplement: Supplementary file 1 — Supplementary file1 (PDF 59 kb) [file 497_2021_418_MOESM1_ESM.pdf]

Table S1. List of plasmid DNA vectors used in this study

Plasmid name	Description	Purpose	Related figure	Reference
DKv327	*35Sp::mTFP1*	Evaluation of promoter activity	Figure 1	This plasmid was provided from Dr. Noriko Inada.
DKv744	*35Sp::H2B-tdTomato*	Evaluation of promoter activity, visualization of bombarded pollen t	Figure 1, 2d, 2e, 2h, 2i, 4a, 4d, 5b-5	This plasmid was provided from Dr. Daisuke Kurihara
sSNv26	*AtUBQ10p::tdTomato*	Evaluation of promoter activity, visualization of bombarded pollen t	Figure 1, 3a, 3c	Constructed in this study
sSNv10	*AtRPS5Ap::sGFP*	Evaluation of promoter activity	Figure 1	Constructed in this study
DKv277	*AtRPS5Ap::H2B-tdTomato*	Evaluation of promoter activity, visualization of bombarded pollen t	Figure 1	Adachi et al, 2011
sSNv28	*AtUBQ10p::H2B-mClover*	Transformation, visualization of bombarded pollen tube	Figure 1, 2a, 2d, 2e, 2g, 2i, 3a, 4a, 4	Constructed in this study
YMv32	*LAT52p::mApple*	Multiple expression, evaluation of delivery efficiency	Figure 2a, 2g, 4d	Mizuta et al., 2015
sSNv21	*AtUBQ10p::Cas9/U6.26p::NbPDS3-sgRNA*	Genome editing in leaf and pollen	Figure 3b, 3d	Constructed in this study
sSNv25	*AtUBQ10p::sGFP*	*N. tabacum* semi-in vivo assay	Figure 4d, 5b-5h	Constructed in this study
