# Supplementary material for: Detection of a biolistic delivery of fluorescent markers and CRISPR/Cas9 to the pollen tube
Source: Plant Reprod. 2021 Jun 19;34(3):191–205. doi: 10.1007/s00497-021-00418-z (PMC8360903; doi:10.1007/s00497-021-00418-z)
Supplement: Supplementary file 2 — Supplementary file2 (PDF 39 kb) [file 497_2021_418_MOESM2_ESM.pdf]

Table S2. List of primers used in this study

Primer name	5' -> 3' sequence	Purpose
DKp113	CCATGGTGAGCAAGGGCGAGGAG	Construction of sSNv10
DKp130	CCATGTCGACGGCTGTGGTGAGAG	Construction of sSNv10
NbPDS3_sgRNA-1_FWD	ATTGCCGTTAATTTGAGAGTCCA	Construction of CRISPR/Cas9 vector targeting NbPDS3
NbPDS3_sgRNA-1_RVS	AAACTGGACTCTCAAATTAACGG	Construction of CRISPR/Cas9 vector targeting NbPDS3
RPS5Ap-R1	AGATGTGATGAACGCCACAG	Construction of modified pKI1.1 vector
pTTK346_inverse_F	CCATGGACTATAAGGACCACGAC	Construction of modified pKI1.1 vector
GA_UBQ10p_FWD	GCGTTCATCACATCTCGACGAGTCAGTAATAAACG	Construction of modified pKI1.1 vector
GA_UBQ10p_RVS	CCTTATAGTCCATGGCTGTTAATCAGAAAAACTCAG	Construction of modified pKI1.1 vector
PDS_MlyIF	GCTTTGCTTGAGAAAAGCTCTC	PCR of NbPDS3 sequence
PDS_MlyIR	ACATAACAAATTCCTTTGCAAGC	PCR of NbPDS3 sequence
NbPDS3_nest_F	TTTTCCCGTTTAGGATCTTG	Nested PCR of NbPDS3 sequence
NbPDS3_nest_R	GCAAACATCTTGACTTTCAG	Nested PCR of NbPDS3 sequence
M13 forward	GTAAAACGACGGCCAGT	Colony PCR to detect mutation in NbPDS3
M13 reverse	CAGGAAACAGCTATGAC	Colony PCR to detect mutation in NbPDS3
NbPDS3 primer-m	GATAAGCTGAATTACCTTGGAC	Colony PCR to detect mutation in NbPDS3
