# Supplementary figures and images for: Detection of a biolistic delivery of fluorescent markers and CRISPR/Cas9 to the pollen tube
Source: Plant Reprod. 2021 Jun 19;34(3):191–205. doi: 10.1007/s00497-021-00418-z (PMC8360903; doi:10.1007/s00497-021-00418-z)

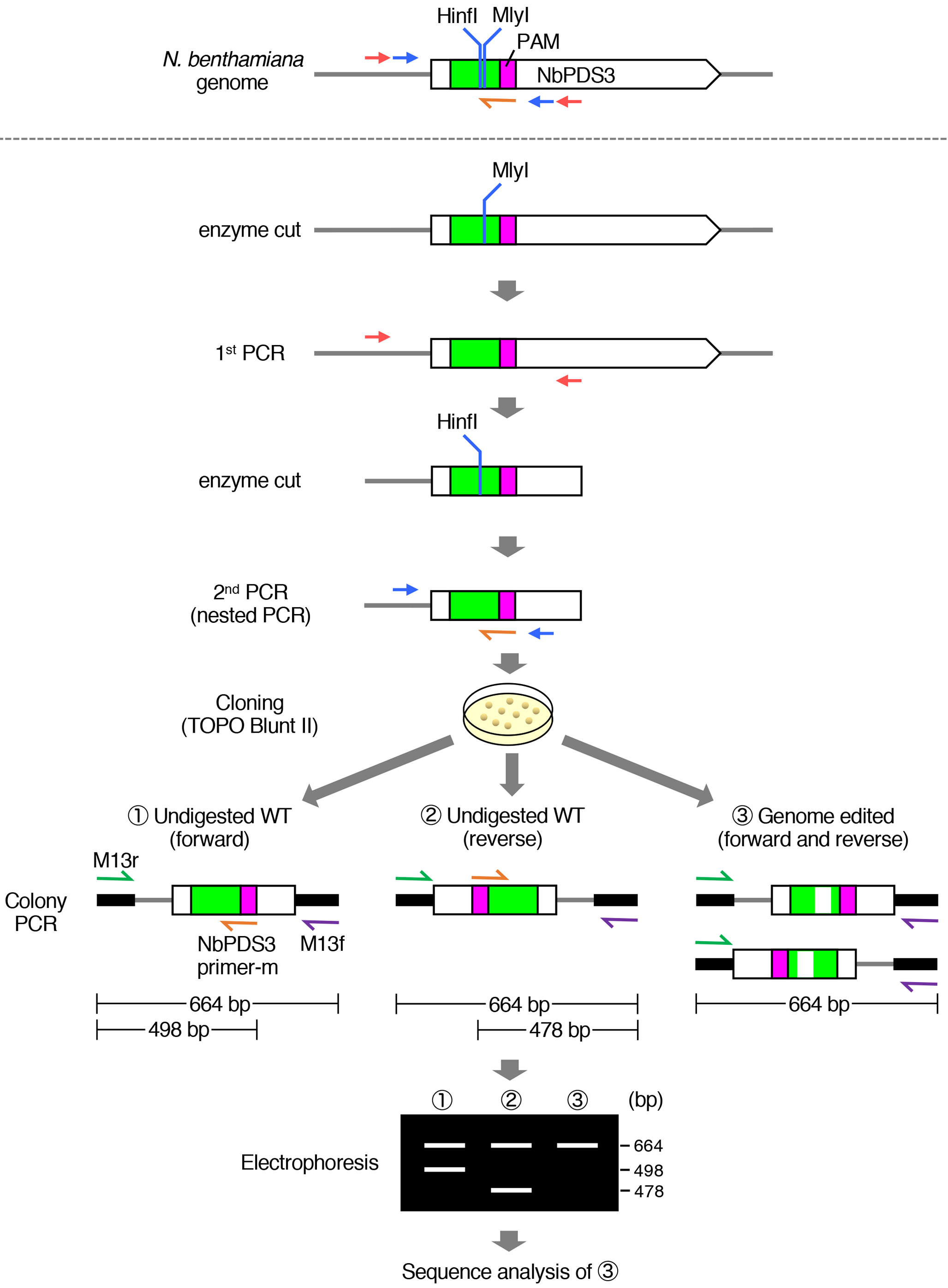

Supplement: Supplementary file 3 — Supplementary file3 (TIF 393 kb) [file 497_2021_418_MOESM3_ESM.tif]
